# Concanavalin A-induced autoimmune hepatitis model in mice: Mechanisms and future outlook

**DOI:** 10.1515/biol-2022-0013

**Published:** 2022-02-28

**Authors:** Yang Liu, Huiqin Hao, Tiezheng Hou

**Affiliations:** College of Basic Medical Sciences, Shanxi University of Chinese Medicine, Jinzhong, 030619, PR China; Basic Laboratory of Integrated Traditional Chinese and Western Medicine, Shanxi University of Chinese Medicine, Jinzhong, 030619, PR China

**Keywords:** autoimmune hepatitis, concanavalin A, experimental animal models, inflammation, pathogenesis

## Abstract

The concanavalin A (Con A)-induced liver injury mouse model is a typical animal model focusing on T cell-dependent hepatic damage in the field of autoimmune hepatitis (AIH). However, the underlying mechanism of hepatic dysfunction due to cell activation or signaling pathways triggered by Con A has not been fully clarified. Therefore, the controversy on this model remains in the academic community. In this article, we first summarized the merit and demerit of this contentious model from the perspectives of cell dysfunction, microcirculation disturbance, involved signaling pathways, as well as the properties of Con A. Then, we summed up the scientific implications of the model in elucidating the pathogenesis of AIH, and the shortcomings of this model were also summarized to elucidate the pathogenesis and application prospect of this classical liver injury mouse model in the study of AIH.

## 1 Introduction

Autoimmune hepatitis (AIH), originally named “active chronic and lupoid hepatitis” by Ian R. Mackay and F. Macfarlane Burnet in 1963, is a type of chronic progressive inflammation of liver parenchyma mediated by autoimmune response [1]. It is closely related to the abnormal proliferation and activation of T lymphocytes and is distinguished by interfacial hepatitis in histology and by elevation of immunoglobulin G level and presence of autoantibodies in serology. Moreover, it eventually may cause fibrosis and cirrhosis if the inflammation process cannot be controlled [2]. AIH occurs globally in all ages and ethnicities with a strong female predominance [3], and its incidence and prevalence are still increasing in recent years [4,5]. Establishing an ideal experimental animal model to mimic the progress of human AIH in the laboratory is thought to be helpful in better studying the specific pathogenesis of this disorder and exploring more effective clinical treatment methods. Many researchers have devoted themselves to the evolution of animal models related to AIH since the early 1970s [6]. Notwithstanding a variety of animal models that have been applied to the study on the etiopathogenesis of AIH in different phases, the concanavalin A (Con A)-induced liver injury mouse model is regarded as the most important one [7,8]. It was successfully established in 1992 by Tiegs G [9] and was commonly used in such research fields from the 1990s to 2020s [10,11,12,13]. Not only this model is easy, convenient, inexpensive, and repeatable to establish [14,15] but also its histological features (including periportal and intralobular lymphocytes infiltrates as well as interface hepatitis) and serological changes (i.e., high levels of transaminase) are similar to human AIH patients [16]. However, this widely applicated mouse model is still debatable with the deepening of research on the mechanisms causing liver injury [17]. An in-depth summary of the controversies over this model, together with its pros and cons, will be conducive to illustrate its status and meet the future challenge in the study of AIH.

## 2 Multiple populations of cells participating in the hepatic injury in this model

**Figure 1:**
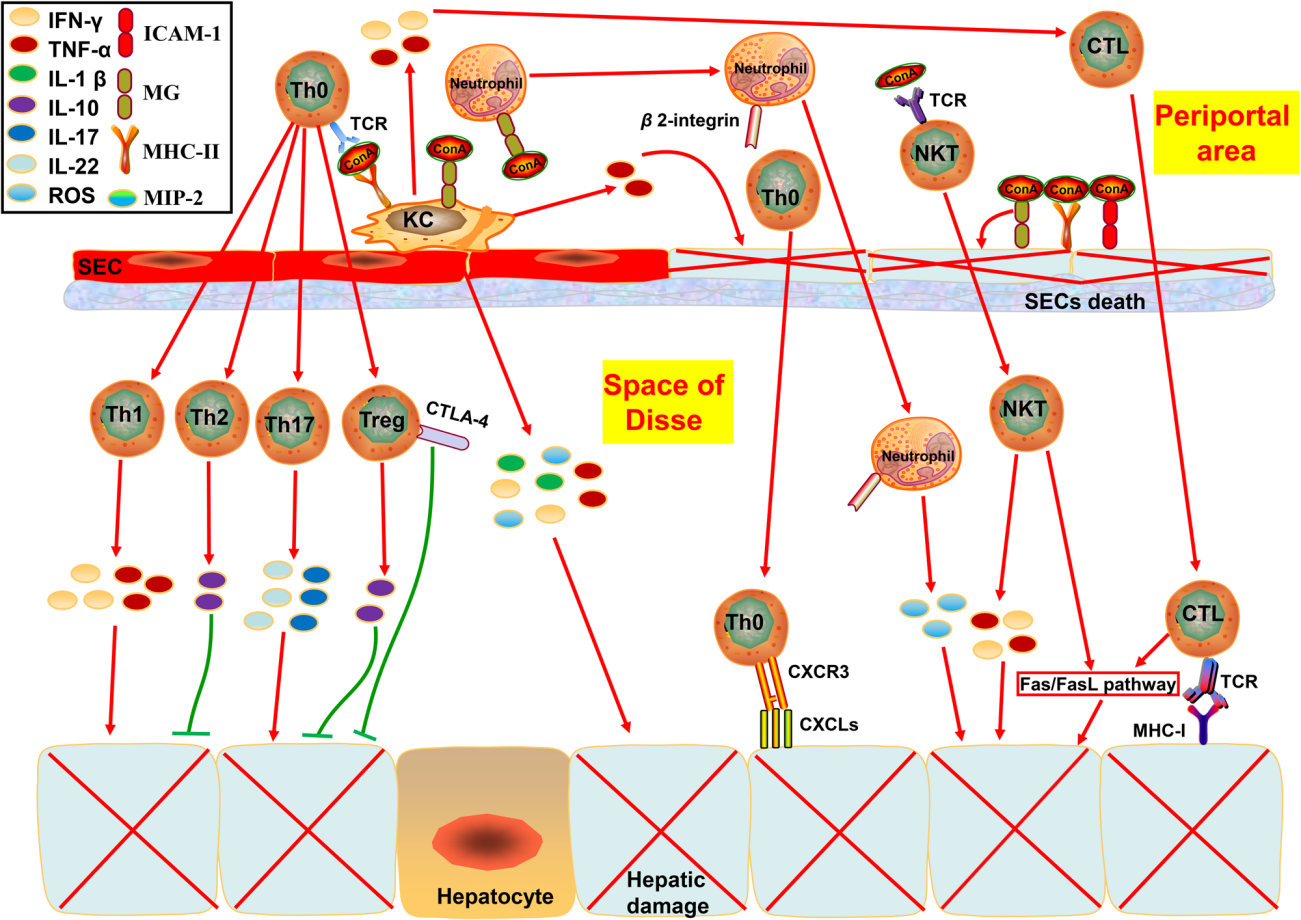
The multicellular participation mechanism participated in establishing the Con A-induced liver injury mouse model. Con A was capable of attaching to MHC class II, MR, and ICMA-1. Th0 cell differentiated into Th1, Th2, Th17, and Treg by the recognition of MHC class II-Con A complex via TCR, and Th1 and Th17 induced the hepatocyte necrosis and apoptosis by secreting TNF-α, IFN-γ, IL-17, and IL-22, while Th2 and Treg would induce tolerance toward Con A by secreting IL-10 or expressing CTLA. Th0 could also migrate to the inflamed tissue by adhering to CXCLs via CXCR3. The NKT directly induced the death of hepatocytes or SECs by expressing TNF-α and IFN-γ after combining with this lectin through TCR. The MRs, located on the surface of macrophages and neutrophils, contain multiple CTLD, and the *N*-acetylglucosamine of Con A can combinate to the MRs to induce the receptor-mediated endocytosis and phagocytosis to maintain the stability of the internal environment. At the same time, Con A also can lead to the activation of macrophages and neutrophils via MRs, to increase the release of superoxide anions and induce the synthesis of cytokines (including TNF-α, IFN-γ, IL-1β, MIP-2, and ROS). Con A promoted the expression of β 2-integrin on neutrophils, which was essential for neutrophil recruitment. The attachment of SECs to Con via MR, MHC class II, and ICAM-1 could give rise to their death, facilitating the adhesion of Con A to KCs. CTLs would activate by the Con A-mediated expression of TNF-α and IFN-γ and infiltrate to the necrotic area by recognizing MHC class I expressed on the surface of damaged hepatocytes, which is absent from the normal liver tissue. Both CTLs and NKTs contributed to the apoptosis of hepatic cells via the Fas/FasL pathway.

Initially, the Con A-induced liver injury mouse model was regarded as a well-established CD4+ T helper cell (Th) activated model focusing merely on the dysfunctional Th involved in the pathogenesis of AIH [9]. However, as the research moved along, it was revealed that, except for Th, a variety of other immunocytes and hepatic nonparenchymal cells were related to the development of hepatocellular injury, including cytotoxic CD8 + T lymphocyte (CTL) [18], natural killer T cell (NKT) [19,20], neutrophil [21], kuffer cell (KC) [22,23], and sinusoid endothelial cell (SEC) [24,25]. As shown in Figure 1, Con A is capable of attaching not only to the major histocompatibility complex (MHC) class II expressed on KC [26] but also to the mannose receptor (MR) located on the surface of SEC [9], KC [27,28], and neutrophil [29]. We have confirmed that the effect on macrophage induced by Con A was related to the MRs by performing a series of *in vivo* experiments (the data have not been reported). The MRs, which are located on the macrophage surface, contain multiple C-type lectin-like domains (CTLD), and the *N*-acetylglucosamine of Con A can combinate to the MRs to induce receptor-mediated endocytosis and phagocytosis to maintain the stability of the internal environment. At the same time, Con A also can lead to the activation of macrophages via MRs, to increase the release of superoxide anions and induce the synthesis of cytokines. These cells participate in hepatic damage in direct or indirect ways. For example, Th0 cells will be activated by recognizing the MHC class II-Con A complex via T cell receptor (TCR) and differentiated into Th1, Th2, Th17, and regulatory T cell (Treg). Th1 and Th17 are characterized by secreting tumor necrosis factor (TNF)-α, interferon (IFN)-γ, and interleukins (IL)-17, IL-22 to induce hepatocyte necrosis and apoptosis [30]. At the same time, Th2 and Treg are featured by secreting IL-10 and expressing cytotoxic T lymphocyte antigen (CTLA)-4, which are supposed to induce tolerance toward Con A-induced liver damage [31]. Th0 can also migrate to the inflamed tissue by the recognition of CXC chemokine ligands (CXCLs) expressed on the cytomembrane of injured hepatocytes via CXC chemokine receptor motif 3 (CXCR3) [32,33]. Although CTL does not have an immediate relationship with Con A, the activization of CTL is deemed to be induced by the Con A-mediated expression of TNF-α and IFN-γ, and CTLs will infiltrate the necrotic area by the recognition of MHC class I expressed on the surface of damaged liver cells, which is absent from the healthy liver tissue [34]. That is why CD4+ T cells are reported to be particularly numerous in periportal areas, while CTLs constitute the major cell type in the area of interface lymphocytic infiltration [35], and the ratio of CD4+ T cell to CTL in Con A-induced AIH mice model is higher than in other liver diseases [36,37]. Both CTLs and NKTs contribute to the apoptosis of hepatic cells via the Fas/FasL pathway [34]. Unlike T cells, the KC, neutrophil, and NKT are able to directly induce the death of hepatocytes or SECs by expressing TNF-α, IFN-γ, IL-1β, macrophage inflammatory protein (MIP)-2, and reactive oxygen species (ROS) after being activated by this lectin through TCR or MR [19]. Con A increases the expression of β 2-integrin on neutrophils as well, and the recruitment of neutrophils is a β 2-integrin-dependent process [38]. Moreover, SECs are capable of binding to Con A at 4 h after intravenous injection via MR, MHC II, and intracellular adhesion molecule (ICAM)-1 on its surface. This attachment will lead to the breakdown of the SEC’s membrane and bleb formation and eventually give rise to their detachment, facilitating the adhesion of Con A to KCs [14]. So numerous cells are involved in constructing this model that it is like getting blood from a stone to improve the hepatic injury by interfering with the abnormal activation of a particular type of cell. It is probably the main reason why this model’s etiopathogenesis is still obscure, and no appropriate and acknowledged *in vitro* model was able to identically imitate the pathogenetic process of this hepatitis model, unlike other autoimmune diseases, such as rheumatoid arthritis [39].

However, this model’s “unexpected” multicellular participation mechanism opens a gate to closely mirror most of the pathogenic properties in AIH human patients [40]. On the other hand, it is because of the abundant “unintended” hepatic nonparenchymal cells (such as SECs and KCs) involved in the establishment of this model that the binding of Con A to the liver seemed to be very specific [41]. It has been demonstrated that the organotropism of FITC-labeled Con A for the liver was remarkable, while fluorescence in other tissues was so weak that it was undetectable, including the lung (the organ that Con A passed first after intravenous injection) and kidney (the organ has the largest blood flow). The selective organ damage induced by Con A results from the MRs significantly expressed on the SECs and KCs, and Con A-inducible lesions were absent in macrophage-depleted animals [26,42]. Therefore, although it is not consistent with the original purpose of establishing this model, it is still widely used now.

## 3 Genetic background and gender preference of animal deserving to be taken into consideration

As more and more experiments were carried out in different mouse strains, including C57BL/6 [43], C3H [44], BALB/c [45], NMRI [46], and FVB/N [47], it was demonstrated that the dosage of Con A needed to trigger hepatic injury varied significantly according to the genetic background of mice. Generally, Th1-biased C57BL/6 and C3H mice are most susceptible to Con A and normally require lower doses of Con A only around 15–20 mg/kg body weight, while mice with Th2-biased immune response, such as BALB/c or NMRI mice, commonly need higher doses of this lectin up to >30 mg/kg body weight [48]. Therefore, the genetic background of animals needs to be considered when establishing this hepatitis model, due to that Con A is prone to induce a Th1-biased immune response.

Though AIH shows the female preponderance with a female-to-male ratio of 4:1 [49], it is preferable to implement the Con A-induced liver injury experiment exclusively using male mice in order to minimize the number of animals required for reaching statistically significant results. The prime reason is that the immune response induced by Con A is considerably dependent on the hormonal state of the animal, and the female mice are more sensitive to Con A than male ones and show a greater variation in the degree of liver injury and level of cytokine production [50]. Nevertheless, it is helpful to study the gender-related differences in the development of human AIH patients by taking advantage of the gender preference of the model.

## 4 Intricate signal transduction pathways involved in the pathogenesis of this model

Along with the deepening of the research, a complicated network of signal transduction pathways referring to provoking or exacerbating the impairment of liver function in this model was gradually disclosed, including “Toll like receptor (TLR) signaling pathway” [51,52], “MAPK signaling pathway” [53], “PI_3_K/Akt signaling pathway” [54,55], “Ferroptosis” [56], “Notch signaling pathway” [57], “Wnt signaling pathway” [58,59], “Endocytosis” [60], and so on. The data in one of our earlier experiments also showed that the differentially expressed mRNAs (DEMs) filtered out by microarray with this model were significantly enriched in these pathways [61]. Furthermore, many new therapeutic antibodies, drugs, or monomers targeting these validated pathways have been increasingly developed to attenuate hepatic damage. For instance, fucosterol and nobiletin were confirmed to alleviate the acute liver lesion through regulating the “MAPK signaling pathway” [62,63], ghrelin and salidroside were deemed to improve liver function in this model via “PI_3_K/Akt signaling pathway” [64,65], while Hu23C3 (a humanized murine monoclonal antibody against human osteopontin) and CpG-containing oligodeoxynucleotides were supposed to mitigate the hepatic injury and enhance the survival rate of mice by interfering with “NF-κB signaling pathway” [66,67]. Nevertheless, the complexity of the interaction of these pathways exceeded the expectation so that the nosogenesis of this model became more and more incomprehensible, and there is still no specific drug that is able to thoroughly improve the inflammatory damage to liver.

**Figure 2:**
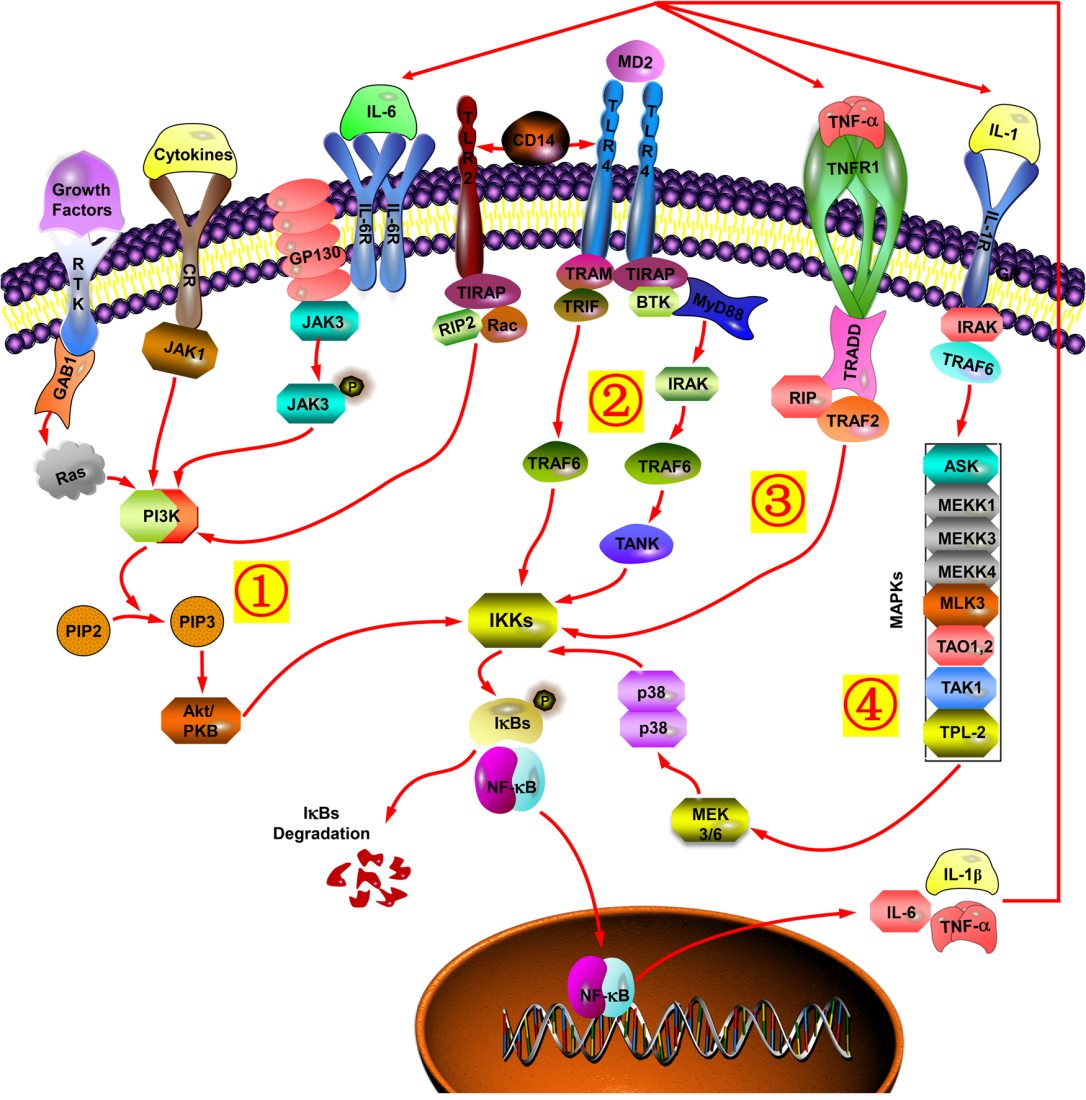
The signal transduction pathways targeting NF-κB are involved in building the Con A-induced AIH mouse model. Numerous inflammatory signals (such as IL-1β, IL-6, TNF-α, and some other pro-inflammatory cytokines) could give rise to the activation of IKKs and translocation of NF-κB dimers from the cytoplasm to the nucleus, through multiple signal transduction pathways, including “TLR signaling pathway”, “MAPK signaling pathway”, and “PI3K/Akt signaling pathway”. The nuclear translocation of NF-κB resulted in the synergistic expression of multiple inflammatory and innate immune genes eventually, involving IL-1β, IL-6, and TNF-α as well. Thus, IL-1β, IL-6, TNF-α, and NF-κB formed an amplifying feed-forward loop. (1) PI3K/Akt signaling pathway, (2) TLR-2 and 4 signaling pathway, (3) TNF-α signaling pathway, and (4) MAPK signaling pathway.

According to the published literature, we found that NF-κB is a vital hub gene associated with the setting up of this hepatitis model because this transcription factor is likely to be the intersection of numerous signal transduction pathways [68,69,70]. Parts of the pathways probably related to NF-κB were exhibited in Figure 2. NF-κB was initially reported as a B-cell-specific transcription factor that binds the κB site in the Igκ light chain enhancer [71]. It can be activated by a variety of inflammatory signals. Pro-inflammatory cytokines and pathogen-associated molecular patterns, working through different receptors, including TNF receptor (TNFR), TLR, and IL-1 receptor (IL-1R) superfamilies, cause the activation of inhibitory κB kinase (IKK) complex and translocation of NF-κB dimers from the cytoplasm to the nucleus, resulting in synergistic expression of multiple inflammatory and innate immune genes eventually. The IL-1β, IL-6, and TNF-α activate NF-κB. Meanwhile, expressions of these pro-inflammatory cytokines are also modulated in response to the activation of NF-κB, thus forming an amplifying feed-forward loop [72,73]. Therefore, drug development targeting this hub gene may be more promising.

## 5 Acute inflammation process and no detectable autoantibodies

Admittedly, AIH starts with an episode of acute hepatitis in some cases. However, most of the patients are typical of progressive chronic hepatitis, and hepatic fibrosis or cirrhosis will ultimately be caused if the autoimmune process is not well-controlled [2,74]. Whereas Con A-induced liver inflammation is typical acute hepatitis. The hepatocyte apoptosis and elevated transaminase levels in serum can be detected as early as 5 h after administration, and it is sufficient to investigate the inflammatory phenotype at 8–12 h after receiving an injection [48]. In addition, it is not feasible to perform long-term follow-up experiments or mimic a chronic inflammatory process through a repetitive application of Con A because mice are able to develop protective immune tolerance against hepatitis induced by this lectin, and IL-10 plays an important role in this course [31]. As time goes by, once Con A is metabolized, the self-repair of the liver will initiate, which can be manifested by declined transaminase levels in serology and hepatocyte regeneration in histology. At last, the pathological process of AIH terminates, and liver damage will be restored. Con A-induced liver damage is transient and unsustainable, so it is not suitable for exploring the pathogenic mechanism of chronic hepatitis.

The presence of specific antibodies to particular liver autoantigens in serum is regarded as one of the core diagnostic criteria of AIH [75,76], including anti-nuclear antibody, anti-smooth muscle antibody, anti-liver-kidney microsomal antibody, and anti-liver cytosol type 1 antibody [77,78]. Nonetheless, no circulating autoantibody is produced in the Con A-induced liver injury mice model. For one thing, it is a lack of requirement for antigen specificity in this model, owing to that Con A is the unique agent to activate and recruit T lymphocytes into the liver tissue [9]. For another, the low levels of IL-4, IL-10, and IL-13 also contribute to the shortage of autoimmune antibodies. Because these cytokines, which are mainly secreted by Th2 cells, are essential for B cells maturation to plasma cells to secrete autoantibodies [2,79]. Under the healthy condition, the ratio of Th1 to Th2 is in dynamic equilibrium and controlled by the transcription factor T-bet and GATA-binding factor type 3 (GATA3) [80]. Con A promotes the skewing of Th1 by increasing the expression of T-bet and suppressing the differentiation of Th2 via decreasing the expression of GATA3 [81]. As mentioned before, the Th1-biased C57BL/6 and C3H mice are most susceptible to Con A-induced liver injury. In contrast, mice with a Th2-biased immune response, such as BALB/c or NMRI mice, require higher doses of Con A. Without the participation of B cells and no generation of autoantibodies are deemed to be the biggest obstacle in translating this model into the investigation on human AIH.

## 6 The side-effect for Con A as a lectin

### 6.1 Microcirculation disturbance

**Figure 3:**
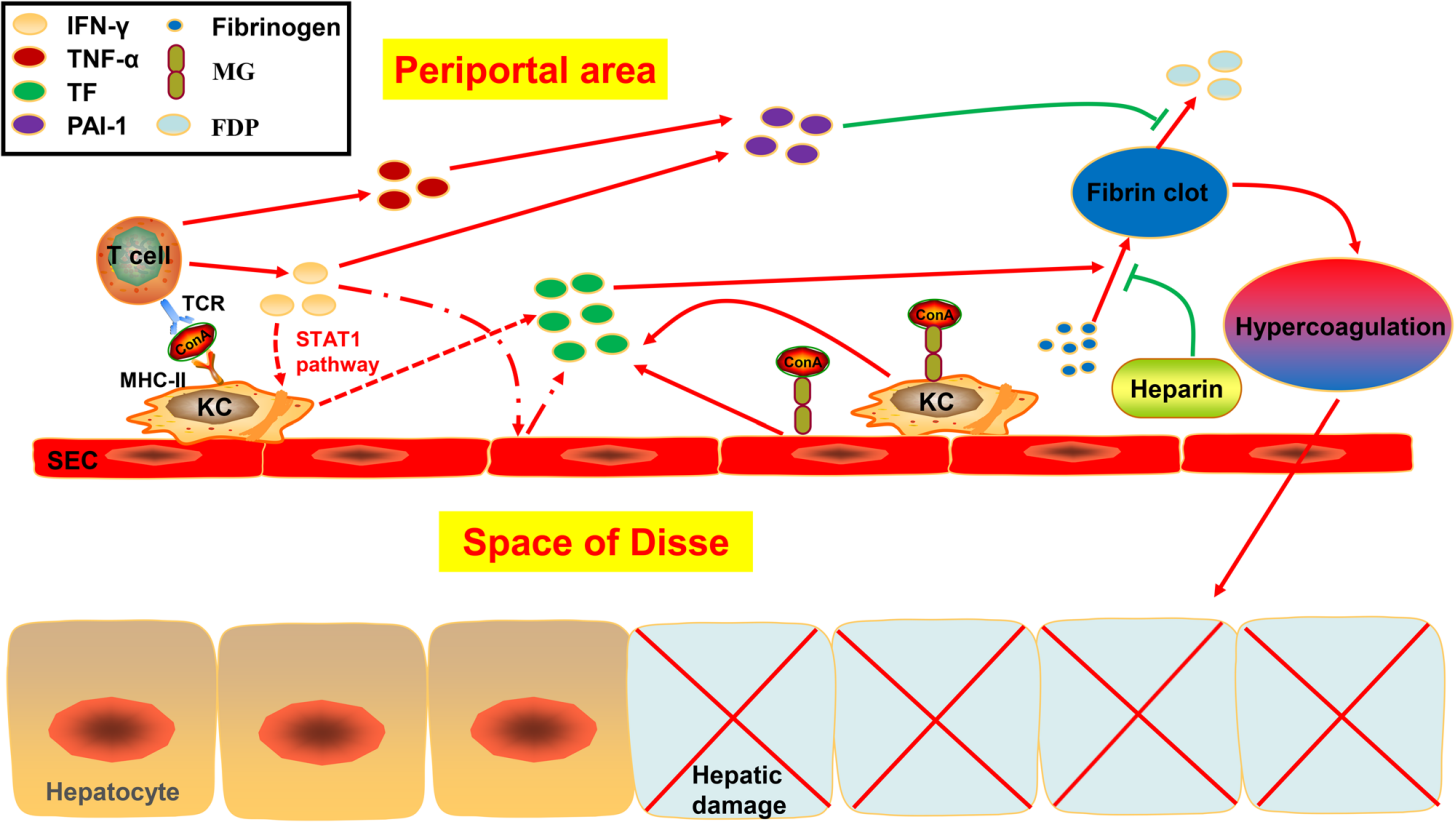
The microcirculation disturbance mechanism related to the foundation of Con A-induced AIH mouse model. Con A first caused a significant decrease in blood flow in the hepatic sinusoid after being administrated. Thereafter, abnormally activated T cells immediately produce a large amount of IFN-γ and TNF-α, and then the STAT1 signaling pathway is activated to promote the SECs and KCs to synergistically express tissue factor (TF). TF facilitated the fibrinogen agglutinating into fibrin clots as a type of starting factor of coagulation reaction. TNF-α triggered the production of plasminogen activator inhibitor type 1 to inhibit the anticoagulant pathway by suppressing the degradation of fibrin clots into fibrinogen degradation product. SECs and KCs could also express TF after adhering to Con A directly. All of them worked together to result in hypercoagulation and form numerous microthrombi, which would eventually contribute to the necrosis of hepatocytes. The anti-clotting agent, such as heparin, has significant protective effects on the hepatic injury induced by lectin through inhibiting the clotting activity.

As previously mentioned, the original intention on the foundation of this hepatitis mouse model was to investigate the T cell-dependent mechanisms of liver injury. Nevertheless, as a lectin with coagulation activity, if administered intravenously, Con A will first cause a significant decrease in blood flow in the hepatic sinusoid rather than hepatocellular injury [82]. After that, SECs, KCs, and abnormally activated T cells will work in coordination to lead to the formation of numerous microthrombi and bring about the necrosis of hepatocytes [83]. The microcirculation disturbance mechanism induced by Con A was visualized in Figure 3, which would explain why it is different to fully interrupt the immunologic injury induced by Con A only depending on the immunosuppressor, and heparin, an important anti-clotting agent with no immunomodulatory function, has significant protective effects on hepatic injury induced by this lectin without reducing the production of IFN-γ and TNF-α [84]. Hence, microcirculation disturbances induced by Con A is an unintended mechanism on the liver damage, and to some extent, it will interfere with the judgment on the outcome of new drugs associated with the immunologic mechanism.

### 6.2 Environmental sensitivity

Since Con A is a water-soluble tetrameric protein complex purified from the crude extract of *Canavalia ensiformis* seeds, its biological activity shows batch-dependent variations [85]. It is sensitive to environmental decay once not stored under protected conditions, so repeated freeze-thaw cycles should be avoided to circumvent protein degradation [86]. Meanwhile, serving as the sole inducer in this model, the levels of transaminase and pro-inflammatory cytokine, considered as the valid index of the severity of the liver injury, change in a Con A-dose-dependent manner [87]. If the intake of Con A is too high, liver damage will be irreversible so that some mice will have the symptom of acute liver failures such as encephalopathy or ascites and die in a short time. Therefore, the dose of Con A needed to induce liver injury has to be tested for each batch before an experimental series, and it is necessary to titrate the Con A dosage accurately to minimize the number of animals succumbing to hypohepatia. Ordinarily, 5–10 mg/kg dose of Con A for 18 h are safe for mice, while 15, 20, and 25 mg/kg will induce 20, 40, and 60% death of mice, respectively [88].

## 7 Application prospect and possible improvements

Although there are some deficiencies in the Con A-induced liver injury mouse model to completely mimic the pathogenesis and pathological process of AIH in human patients, it is still a well-established and easy to replicate animal model focusing on T cell-mediated hepatic damage (Table 1). In addition, this model is also of great significance to develop new therapeutic drugs or other treatment measures just targeting T cell activation. To cover the model shortage, the following improvements could be taken into account to implement.

**Table 1:** The most distinctive features of the Con A-induced AIH mouse model

Features	Refs.
Kinds of cells participating	
CD4 + T cell	[9,30,31,32]
CTL	[18,34,35]
NKT	[19,20,34]
Neutrophil	[19,21,38]
KC	[19,22,23,26,27,28]
SEC	[14,24,25,42]
Male Th1-biased mice are more suitable for establishing this model	[48,50]
Intricate signal transduction pathways involved in the development of this model	[51,52,53,54,55,56,57,58,59,60,61]
Acute inflammation process	[2,31,48,74]
No detectable autoantibodies	[2,9,79,80,81]
Microcirculation disturbance as a lectin	[82,83,84]
Environmental sensitivity	[85,86,87,88]

First, high-throughput detection techniques on screening the differentially expressed genes (involving the coding RNA and non-coding RNA) can be applied to this model, just like the research we are doing, to further explain the interaction between signal transduction pathways [61,89,90]. In particular, much more attention should be paid to the differentially expressed genes enriched in signaling pathways associated with T cell activation and NF-κB transcription.

Second, some kinds of *in vitro* models could be considered to verify the experimental results obtained from this Con A related *in vivo* model. For example, the Con A-mediated macrophage activation model is conducive to further interpret the role of KCs in the pathogenesis of AIH, which are primarily found in mice experiments [91,92]. Due to the dominant role in the development of AIH played by Th1-biased immune response, the Th1 cell *in vitro* model established in accordance with the Th1-skewing condition will serve the purpose of investigating the effects of Th1 cells on liver injury [93,94].

Third, it seems that the utilization of transgenic animals will make up its defects to some extent and is helpful in the discovery of novel biomarkers and potentially new therapeutic targets [95,96,97,98,99].

Furthermore, in view of the agglutination activity of Con A as a lectin, certain kinds of anticoagulant drugs, such as heparin, can be considered to prophylactically administrate to as far as possible mitigate the effects of microcirculation disturbance on the experiments principally aiming at exploring the mechanism of immune disorders.

To sum up, parts of the mechanisms of hepatotoxicity by Con A have been revealed in this article, i.e., Con A, as a kind of lectin, can lead to the activation of macrophage and neutrophil via MRs to increase the release of superoxide anions and induce the synthesis of cytokines, as well as activate Th0 cell via TCR by forming the MHC class II-Con A complex. Con A can also promote the formation of hypercoagulation and form numerous microthrombi in the hepatic sinusoid, which would eventually contribute to hepatocyte necrosis. Moreover, numerous inflammatory signals can be activated after the administration of Con A. Therefore, there is a reasonable prospect that, with continuous improvement and optimization, the research related to the Con A-induced liver injury mouse model will achieve a significant breakthrough and innovation, which will be conducive to a further study in the field of AIH in the future.
